# Survey of potential fungal antagonists of Coffee Leaf Rust (*Hemileia vastatrix*) on *Coffea arabica* in Hawai‘i, USA

**DOI:** 10.1007/s42770-024-01304-2

**Published:** 2024-05-14

**Authors:** Blaine C. Luiz, Lionel S. Sugiyama, Eva Brill, Lisa M. Keith

**Affiliations:** Tropical Plant Genetic Resources and Disease Research Unit, USDA Agricultural Research Service, Hilo, HI 96720 USA

**Keywords:** Coffee, Coffee leaf rust, *Hemileia Vastatrix*, Mycoparasite, Biological control, *Pucciniales*

## Abstract

*Hemileia vastatrix*, causal agent of coffee leaf rust (CLR), is an aggressive pathogen of coffee plants worldwide. Conventional fungicides play a major role in the suppression of this disease, but a recent shift toward eco-friendly farming practices has occurred and additional novel, effective, and sustainable strategies for CLR control are needed. Naturally occurring fungal antagonists could be well-positioned to meet this demand, but these fungi need to be isolated and tested for efficacy to identify organisms with potential. In this study, a survey of fungi associated with CLR lesions in four districts of Hawai‘i Island, HI, USA (Kona, Ka‘ū, Hāmākua, and Hilo) was conducted. Coffee leaves infected with CLR were collected from 22 locations and over 600 lesions were plated on ½ APDA and CTC 4T media. DNA was extracted from purified isolates and the internal transcribed spacer region (ITS) was sequenced and analyzed by BLASTn. In total, 194 isolates comprising 50 taxa were recovered. Several of the genera are known antagonists of CLR or other plant pathogens, including *Simplicillium*, *Akanthomyces*, *Cladosporium*, *Fusarium*, and *Clonostachys*. The wide diversity of fungi associated with CLR lesions provide a wealth of possibilities for identifying potential CLR antagonists that could serve as a valuable tool for coffee farmers as part of an integrated pest management plan.

## Introduction

Coffee leaf rust (CLR) is caused by an obligate parasitic fungus (*Hemileia vastatrix*) that infects the leaves of coffee (*Coffea sp.*) and causes considerable economic losses for farmers in all coffee growing regions [1]. In late 2020, coffee leaf rust was detected in Hawai‘i, which was the last coffee-growing region free of CLR in the world [2]. Since then, CLR has been identified on the six major islands where coffee is grown, including Hawai‘i Island, Maui, O‘ahu, Lāna‘i, Moloka‘i, and Kaua‘i [3, 4]. The origin of the outbreak is unknown; however, isolates recovered locally have been identified as Race XXIV [3] and are most genetically similar to genotypes found in Central America and Jamaica [4]. Due to the recent nature of this detection and Hawai‘i being the primary coffee producing state in the United States, there are few fungicides registered for use that farmers can employ to combat the disease and most of them are contact fungicides or prophylactics [5]. Additionally, applying fungicides to target CLR is a significant economic burden for farmers, which has been estimated as high as 11% of profits [6]. Importation of resistant coffee varieties, one of the best methods for controlling CLR as part of an IPM strategy, is restricted by regulations that limit the continental United States as the origin of the shipment followed by a one-year quarantine [4]. These logistical and financial challenges make it difficult for coffee farmers in Hawai‘i to control CLR.

Microbial antagonists of *H*. *vastatrix* could serve as a valuable tool in a farmer’s arsenal for controlling CLR [7]. Fungal endophytes are of particular interest when surveying for antagonists [8], which can secrete toxic metabolites capable of inhibiting their host pathogens [9, 10]. Additional antagonistic actions include parasitism, competition, and antibiosis. Historically, studies on mycoparasites of CLR have focused on the “white-halo fungi” originally believed to be from the genus *Lecanicillium* [11, 12]. This group of fungi has undergone taxonomic revision, which has separated many of these fungi into different genera including *Akanthomyces* and *Simplicillium* [13, 14]. This finding has allowed for more comprehensive analyses of these fungi as predators of CLR [15–18]. Fungi in other genera have also been discovered to be antagonistic to CLR [19–21], suggesting that broad surveys for potential antagonists of CLR will be fruitful.

Biological control is often a key component of organic farming systems but can be an equally important part of an integrated pest management strategy used in general coffee cropping systems. Due to the limited repertoire of effective CLR control measures in Hawai‘i, locally isolated antagonistic fungi have the potential to be an organic supplement to current control recommendations. Thus, the aim of this study was to survey coffee farms on Hawai‘i Island, HI, USA, one of the main coffee producing islands in the state, for fungi associated with CLR lesions and urediniospores to identify potential CLR-specific antagonists.

## Materials and methods

### Sample collection

Collections of leaf samples infected with *H. vastatrix* spores were conducted at 22 sites in the Kona (14 sites), Ka‘ū (4 sites), Hāmākua (1 site), and Hilo (3 sites) districts of Hawai‘i Island, Hawai‘i, USA from January 2021 to January 2023 (Table 1). All sites were actively managed coffee farms except for sites 19 and 22, which were a feral coffee stand located in a riparian forest comprised of non-native tree species and a homeowner’s backyard coffee plant, respectfully. Leaves were collected by hand and placed between layers of paper towels in a resealable plastic bag. Leaves containing lesions overgrown with mycelium (a potential indication of mycoparasite infection) were prioritized for collection. In the absence of this scenario, any leaves with actively sporulating rust infections were collected. Once collections at a site were completed, the filled plastic bags were placed in a cooler with a cold pack. A sheet of polystyrene foam or cardboard was placed between the plastic bag and the cold pack to keep the leaf samples from directly contacting the cold pack.

**Table 1:** Summary CLR-infected coffee leaf collection sites

Site #	Location	District	Approximate elevation (m)	Leaves collected¹	Lesions sampled¹
1	Hōlualoa	Kona	450	-	-
2	Hōlualoa	Kona	515	-	-
3	Hōlualoa	Kona	454	14	38
4	Hōlualoa	Kona	400	14	44
5	Hōlualoa	Kona	425	26	78
6	Nāpōʻopoʻo	Kona	300	13	16
7	Hōnaunau	Kona	285	20	60
8	Hōnaunau	Kona	488	-	-
9	Hōnaunau	Kona	204	14	42
10	Makalei	Kona	488	15	33
11	Captain Cook	Kona	474	9	27
12	Captain Cook	Kona	607	10	30
13	Captain Cook	Kona	500	4	16
14	Hōnaunau	Kona	452	8	23
15	Pāhala	Kaʻū	689	10	30
16	Pāhala	Kaʻū	360	1	3
17	Pāhala	Kaʻū	472	4	10
18	Pāhala	Kaʻū	600	5	15
19	Paʻauilo	Hāmākua	360	12	36
20	Wainaku	Hilo	155	21	59
21	Pana‘ewa	Hilo	190	10	30
22	Waiākea	Hilo	128	6	18

^1^Number of leaves and lesions sampled not recorded for sites 1, 2, 8, and partially recorded for sites 4 and 17

### Fungal isolation

Isolations were conducted in a biosafety cabinet rated for biosafety level 2 (Labconco, Kansas City, MO, USA) at the USDA ARS Daniel K. Inouye U.S. Pacific Basin Agricultural Research Center (PBARC) in Hilo, HI. Coffee leaf rust pustules with urediniospores were scraped with a sterile scalpel with a #10 blade and streaked on selective agar. From January-September 2021, CTC 4T agar (PDA plus 1 g/l yeast extract supplemented with 500 mg/l chloramphenicol, 4 mg/l thiabendazole, and 250 mg/l cycloheximide) was used for isolation [22]. This agar, which is selective for entomopathogenic fungi, was initially used since many of the well-known parasites of CLR belong to the family Cordycipitaceae [15, 17]. Half-strength potato dextrose agar amended with lactic acid (9.75 g PDA, 3.75 g agar, 500 ml distilled water, 500 µl 85% lactic acid after autoclaved media has cooled) (½ APDA) and CTC 4T agar were used for isolations from December 2021 to January 2023 to broaden the scope of the survey. Cultures were incubated in plastic bins in the laboratory (~ 23 °C) for 4–7 days. The resulting fungal colonies were transferred by single or mass hyphal tips to full-strength PDA and incubated under the same conditions.

### DNA extraction, amplification, sequencing, and isolate identification

Fungal isolates were identified via molecular sequence analysis. Cultures that were 7–22 days old were scraped with flocked-tip swabs (FLOQSwabs, Copan Diagnostics Murrieta, CA, USA) placed in 2 ml homogenization tubes filled with 6 mm zirconium beads. Samples were extracted using the NucleoSpin Plant II mini kit (Macherey-Nagel, Allentown, PA) using the manufacturers specifications and PL1 lysis buffer. To identify the isolates, the internal transcribed spacer (ITS) region was amplified via polymerase chain reaction using primer set ITS5/ITS4 [23] and the following cycling parameters: initial denaturation of 2 min at 95 C; followed by 40 cycles of 30 s at 95 C, 30 s at 55 C, and 30 s at 72 C; final extension of 5 min at 72 C. Successful PCR amplicons were determined by gel electrophoresis and visualized on a 1% agarose gel under UV illumination. PCR products were cleaned-up using ExoSAP-IT (Thermofisher) and prepared and submitted for bulk Sanger sequencing (Eurofins Genomics, Louisville, KY). Sequencing data was analyzed in Geneious Prime (Boston, MA).

Fungal identification was conducted via BLAST (http://www.ncbi.nlm.nih.gov/BLAST) using parameters adapted from Vega et al. [24]. The top 100 matches with a sequence identity of 97% or higher were analyzed for each sequence and isolates were identified to genera based on a consensus of the BLAST results. If the search results were mixed, the most specific taxonomic unit shared by the two most abundant matches in the top 100 closest matches was reported as the identity of the isolate. Isolates identified solely via sequence data were not identified down to the species level out of caution. Fungal isolate sequence data was deposited into GenBank (Accession Nos. OQ878295-OQ878344, see Table 2).

**Table 2:** Fungal isolates recovered from coffee leaf rust lesions originating from Hawai‘i Island, HI, USA

		District			
Fungal ID	GenBank accession number	Kona	Ka‘ū	Hilo/Hāmākua	Total
*Akanthomyces* sp.	OQ878295	0	0	3	3
*Apiospora* sp.	OQ878296	0	1	0	1
Basidiomycota sp.	OQ878297	2	1	1	4
*Beauveria* sp.	OQ878298	3	0	0	3
Capnodiales sp.	OQ878299	0	3	5	8
*Chaetothyriales* sp.	OQ878300	0	0	1	1
*Cladosporium* sp.	OQ878301	35	3	13	51
*Clonostachys* sp. 1	OQ878302	0	0	1	1
*Clonostachys* sp. 2	OQ878303	0	0	1	1
*Colletotrichum* sp.	OQ878304	2	1	1	4
Cordycipitaceae sp. 1	OQ878305	0	5	1	6
Cordycipitaceae sp. 2	OQ878306	3	1	0	4
*Cryptococcus* sp.	OQ878307	0	0	1	1
*Curvularia* sp. 1	OQ878308	1	0	0	1
*Curvularia* sp. 2	OQ878309	0	0	1	1
*Epicoccum* sp. 1	OQ878310	1	0	0	1
*Epicoccum* sp. 2	OQ878311	4	1	0	5
*Epicoccum* sp. 3	OQ878312	0	0	1	1
*Fusarium* sp. 1	OQ878313	3	1	0	4
*Fusarium* sp. 2	OQ878314	1	1	0	2
*Fusarium* sp. 3	OQ878315	1	0	0	1
Hypocreales sp.	OQ878316	1	0	0	1
Hypocreomycetidae sp.	OQ878317	0	0	1	1
*Hyweljonsia* sp.	OQ878318	0	1	0	1
*Meira* sp.	OQ878319	0	0	1	1
*Meyerozyma* sp.	OQ878320	1	0	0	1
*Microdochium* sp.	OQ878321	0	0	1	1
*Nigrospora* sp.	OQ878322	0	0	1	1
*Papiliotrema* sp.	OQ878323	0	1	0	1
*Penicillium* sp. 1	OQ878324	1	0	0	1
*Penicillium* sp. 2	OQ878325	2	0	0	2
*Penicillium* sp. 3	OQ878326	0	0	1	1
*Pestalotiopsis* sp.	OQ878327	0	0	1	1
*Phaeoacrimonium* sp.	OQ878328	1	0	0	1
*Phaeosphaeria* sp. 1	OQ878329	0	1	0	1
*Phaeosphaeria* sp. 2	OQ878330	0	1	0	1
*Phoma* sp.	OQ878331	1	0	0	1
*Pleurodesmospora* sp.	OQ878332	2	0	0	2
*Ramichloridium* sp.	OQ878333	0	0	2	2
*Rhodosporidiobolus* sp.	OQ878334	1	0	0	1
*Simplicillium* sp. 1	OQ878335	1	3	0	4
*Simplicillium* sp. 2	OQ878336	1	0	11	12
*Simplicillium* sp. 3	OQ878337	17	2	19	38
*Simplicillium* sp. 4	OQ878338	4	3	1	8
*Stagonosporopsis* sp.	OQ878339	0	1	0	1
*Stephanonectria* sp.	OQ878340	0	0	1	1
*Talaromyces* sp.	OQ878341	0	0	1	1
*Tinctoporelllus* sp.	OQ878342	0	0	1	1
*Vishniacozyma* sp.	OQ878343	1	0	0	1
*Zasmidium* sp.	OQ878344	0	0	1	1
Total		90	31	73	194

## Results

Over 600 CLR lesions from more than 200 coffee leaves were sampled from Hawai‘i Island. In total, 194 isolates were recovered (Table 2). These isolates assorted into 50 distinct taxa: 44 to genera, one to family, one to order, one to subclass, and one to division. Fungi were isolated from CLR lesions at all sites. The median number of isolates recovered at a given site was 6.5, and Site 20 had the highest taxa richness (15 taxonomically unique isolates). The two most-isolated genera were *Simplicillium* (62 isolates) and *Cladosporium* (51 isolates). All *Cladosporium* isolates were assigned to the taxon “*Cladosporium sp.*” because they did not assort into distinct genotypic groups when the sequences were compared. Seventy-eight isolates were recovered on CTC 4T medium and 116 isolates were recovered on ½ APDA. All taxa were recovered on ½ APDA, while CTC medium was preferred by Cordycipitaceae, some yeasts, and some of the *Cladosporium* isolates.

## Discussion

Many of the isolates recovered in this survey are suspected endophytes in coffee leaves and have been isolated previously in Columbia, Mexico, Puerto Rico, and Hawai‘i [24]. Interestingly, Vega et al. [24] recovered an abundance of *Colletotrichum* species from coffee leaves in Hawai‘i, totaling over 40% of their isolates. In the present study, only four *Colletotrichum* isolates were recovered (2% of the total). *Simplicillium* and *Cladosporium* were the two most isolated genera in this study. The abundance of *Simplicillium* in the present survey may be due to the selection of CLR infected leaves with the presence of mycoparasites and the use of CTC 4T medium solely in the first eight months of the survey. *Cladosporium* species were recovered in both surveys, though to a greater degree in the present survey (21 vs. 3 isolates collected by Vega et al. [24]). Additionally, some of the differences in taxa between the studies could be due to the lack of plant surface sterilization in the present study, which would exclude some surface-dwelling fungi that can also assume an endophytic lifestyle [25]. However, the lack of surface sterilization does not exclude fungi that are strictly epiphytic.

Several taxa that were recovered in the present study may potentially help mitigate CLR through various means of antagonism. Species of *Simplicillium* [16] and *Cladosporium* [26, 27], which were isolated in this study, are known to express antifungal VOCs. Several genera that were recovered are recognized as parasites of CLR including *Simplicillium*, *Akanthomyces, Pleurodesmospora* [28], and *Clonostachys* [29]. Also of interest are *Fusarium* spp., which have been shown to reduce urediniospore germination and disease severity on leaf disks, though mechanisms for antagonism are poorly understood [30]. Isolates from these genera will be prioritized for future efficacy studies against CLR.

The use of CLR-antagonistic fungi as an additional tool could be helpful in the hands of farmers, but further research is needed to confirm this hypothesis on coffee [7]. Testing the efficacy of these organisms against CLR via in vitro germination studies, greenhouse tests with coffee seedlings, and, eventually, field trials will be paramount. Developing a pipeline for conducting this type of research would greatly increase the timeliness and efficiency of producing results. Research into compatibility with host tissues at high spore concentrations will be conducted to identify any constraints in the application of certain CLR-antagonistic taxa. Future studies in Hawai‘i will also focus on continued isolation of potential CLR-antagonistic microorganisms from additional coffee-producing regions in the state.

## Data Availability

All data supporting the findings of this study are available within the paper.
