# Correction: Survey of potential fungal antagonists of Coffee Leaf Rust (*Hemileia Vastatrix*) on *Coffea arabica* in Hawai‘i, USA

**DOI:** 10.1007/s42770-024-01393-z

**Published:** 2024-06-17

**Authors:** Blaine C. Luiz, Lionel S. Sugiyama, Eva Brill, Lisa M. Keith

**Affiliations:** Tropical Plant Genetic Resources and Disease Research Unit, USDA Agricultural Research Service, Hilo, HI 96720 USA

**Correction: Brazilian Journal of Microbiology**

10.1007/s42770-024-01304-2

The copyright holder for this article was incorrectly given as ‘This Agreement will be governed by the federal laws of the United States of America without regards to any conflict of law provisions 2024’ but should have been ‘This is a U.S. Government work and not under copyright protection in the US; foreign copyright protection may apply 2024’.

The original article has been corrected.

